# Vasoregression Linked to Neuronal Damage in the Rat with Defect of Polycystin-2

**DOI:** 10.1371/journal.pone.0007328

**Published:** 2009-10-06

**Authors:** Yuxi Feng, Yumei Wang, Oliver Stock, Frederick Pfister, Naoyuki Tanimoto, Mathias W. Seeliger, Jan-Luuk Hillebrands, Sigrid Hoffmann, Hartwig Wolburg, Norbert Gretz, Hans-Peter Hammes

**Affiliations:** 1 5th Medical Department, Medical Faculty Mannheim, University of Heidelberg, Mannheim, Germany; 2 Ocular Neurodegeneration Research Group, Centre for Ophthalmology, Institute for Ophthalmic Research, University of Tuebingen, Tuebingen, Germany; 3 Department of Pathology & Laboratory Medicine, Division of Pathology, University Medical Center Groningen, University of Groningen, Groningen, the Netherlands; 4 Medical Research Center, Medical Faculty Mannheim, University of Heidelberg, Mannheim, Germany; 5 Institute of Pathology, University of Tuebingen, Tuebingen, Germany; Universidad Peruana Cayetano Heredia, Peru

## Abstract

**Background:**

Neuronal damage is correlated with vascular dysfunction in the diseased retina, but the underlying mechanisms remain controversial because of the lack of suitable models in which vasoregression related to neuronal damage initiates in the mature retinal vasculature. The aim of this study was to assess the temporal link between neuronal damage and vascular patency in a transgenic rat (TGR) with overexpression of a mutant cilia gene polycystin-2.

**Methods:**

Vasoregression, neuroglial changes and expression of neurotrophic factors were assessed in TGR and control rats in a time course. Determination of neuronal changes was performed by quantitative morphometry of paraffin-embedded vertical sections. Vascular cell composition and patency were assessed by quantitative retinal morphometry of digest preparations. Glial activation was assessed by western blot and immunofluorescence. Expression of neurotrophic factors was detected by quantitative PCR.

**Findings:**

At one month, number and thickness of the outer nuclear cell layers (ONL) in TGR rats were reduced by 31% (p<0.001) and 17% (p<0.05), respectively, compared to age-matched control rats. Furthermore, the reduction progressed from 1 to 7 months in TGR rats. Apoptosis was selectively detected in the photoreceptor in the ONL, starting after one month. Nevertheless, TGR and control rats showed normal responses in electroretinogram at one month. From the second month onwards, TGR retinas had significantly increased acellular capillaries (p<0.001), and a reduction of endothelial cells (p<0.01) and pericytes (p<0.01). Upregulation of GFAP was first detected in TGR retinas after 1 month in glial cells, in parallel with an increase of FGF2 (fourfold) and CNTF (60 %), followed by upregulation of NGF (40 %) at 3 months.

**Interpretation:**

Our data suggest that TGR is an appropriate animal model for vasoregression related to neuronal damage. Similarities to experimental diabetic retinopathy render this model suitable to understand general mechanisms of maturity-onset vasoregression.

## Introduction

The retina is a highly vascularized neural tissue. Diabetic retinopathy is characterized by progressive vasoregression which is initiated by pericyte loss [1], [2]. Subsequent loss of endothelial cells leads to formation of acellular capillaries. Finally, retinal vessels undergo vasoregression.

Diabetic retinopathy is not only a microangiopathy, but also a disease involving neurons and glial cells. Recent evidence suggests that neuronal dysfunction and glial changes precede or parallel vascular damage in diabetic retinopathy [3]. Dysfunction of neuronal cells has been reported in diabetic retinas without the occurrence of vascular damage both in experimental diabetes models as well as in patients [4], [5], [6], [7], [8]. Damages to the neurons in diabetic retinas can be attributed to activation of glial cells before the onset of overt vascular abnormalities [9], [10]. The glial cells in the retina, astrocytes and Müller cells, closely interact with the retinal vasculature through their end feet enwrapping vessels. Protective growth and trophic factors, such as vascular endothelial factor (VEGF), basic fibroblast growth factor (FGF2), ciliary neutrophic factor (CNTF) and nerve growth factor (NGF) can be induced in glial cells by several stimuli [11], [12], [13]. In diabetic retinopathy, the interaction between neurons, glial cells and the vasculature is complex and the precise mechanisms between different systems remain poorly understood.

In the streptozotocin-diabetic retinopathy model, neuronal and vascular changes are commonly mild, producing conflicting results about neuronal dysfunction and their relation to vascular changes. In models of retinal dystrophy, neuronal changes are strong, but they occur when retinal vessels have not yet fully developed [14], [15]. Therefore, they cannot reflect the impact of neuronal damage after vessel maturation.

Several studies have demonstrated that ciliopathy, such as polycystic kidney disease correlated with polycystins, is associated with retinal degeneration [16], [17], [18]. Structurally, cilia are localized in the connecting part between the outer and the inner segment of photoreceptor cells [19]. Although occasionally observed in ciliopathy, the consequences of neuronal death and the functional role of neurons in vessel survival and physiology following photoreceptor damage are largely unknown.

Using a transgenic rat (TGR) with overexpression of the mutant cilia gene polycystin-2, we sought to investigate the spatial and temporal development of neuronal degeneration and its functional consequences on the mature retinal vasculature. We used quantitative retinal morphometry, markers of apoptosis, electroretinogram, and expression analysis of neurotrophic and angiogenic growth factors. To this end, we established a clear temporal relationship between neuronal degeneration, glial activation and vessel regression and found a predominance of neurotrophic factor activation.

## Materials and Methods

### Animals

Generation of the TGR in which truncated human polycystin-2 is under the control of CMV promoter, has been described previously [16]. Male homozygous transgenic rats (transgenic line TGR 247) were used in this study and male Sprague-Dawley (SD) rats served as control. The rats were housed in a 12 h light/dark cycle and were allowed free access to food and drinking water. The study was carried out in TGR and SD rats at 1, 2, 3, 5 and 7 months. After sacrifice, eyes were enucleated and immediately fixed in 4% formalin for histological analysis or immediately frozen at −80°C for further analysis.

### Ethics Statement

The Study was performed in accordance with the ARVO statement for the use of animals in ophthalmic and vision research. All animal work was approved by the appropriate committee (Regierungspräsidium, Karlsruhe, Germany).

### Histological analysis

Eyes fixed in 4% formalin over night were embedded in paraffin. 3-micrometer vertical sections around optic nerves were selected to be dewaxed and rehydrated, then stained with Periodic Acid Schiff's (PAS) and hematoxylin according to standard histological staining protocols. Analysis of thickness and number of cells in retinal nuclear layers was performed at the inner border of peripheral 1/3 retina by using a microscope (Leica DM RBE, Bensheim, Germany) equipped with an analysis program (Leica IM50, Herrbrugg, Switzerland). Thickness of the entire retina, the ganglion cell layer, the inner nuclear layer and the outer nuclear layer was measured and number of cells in the ganglion cell layer, the inner nuclear layer and the outer nuclear layer were counted and expressed in each 100 micrometer retina length.

### Terminal transferase dUTP nick-end labeling (TUNEL)

Detection of DNA strand break in apoptotic cells in the retina was identified using TUNEL labeling according to the manufacturer's instructions (Roche, Mannheim, Germany). Briefly, 6 micrometer paraffin sections were dewaxed, rehydrated and treated with 0.025 % trypsin for 2 min. Sections were then incubated with TUNEL reaction mixture containing TdT and fluorescencein dUTP, followed by counter staining with DAPI (4,6-diamidino-2-phenylindole). The apoptotic cells were visualized with a fluorescence microscope (Leica BMR, Bensheim, Germany).

### Electroretinogram (ERG)

Electroretinograms were performed according to previously described procedures [20]. The ERG equipment consisted of a Ganzfeld bowl, a direct current amplifier, and a PC-based control and recording unit (Multiliner Vision; VIASYS Healthcare GmbH, Hoechberg, Germany). Rats were dark-adapted overnight and anaesthetised with ketamine (100 mg/kg body weight) and xylazine (5 mg/kg body weight). The pupils were dilated and single flash ERG recordings were obtained under dark-adapted (scotopic) and light-adapted (photopic) conditions. Light adaptation was accomplished with a background illumination of 30 candela (cd) per square meter starting 10 minutes before recording. Single white-flash stimulus intensity ranged from −4 to 1.5 log cd*s/m^2^ under scotopic and from −2 to 4 log cd*s/m^2^ under photopic conditions, divided into 10 and 8 steps, respectively. Ten responses were averaged with an inter-stimulus interval (ISI) of either 5 seconds (for −4, −3, −2, −1.5, −1, and −0.5 log cd*s/m^2^) or 17 seconds (for 0, 0.5, 1, and 1.5 log cd*s/m^2^).

### Immunofluorescence

6 micrometer paraffin sections were deparaffinized, rehydrated, microwaved in citrate buffer for 20 min, and then treated with 1 % BSA and 0.5 % Triton-100 in PBS. Subsequently, sections were incubated with rabbit anti glial fibrillary acidic protein (GFAP) at a concentration of 1∶200 (DAKO Cytomation, Hamburg, Germany) at 4°C overnight. After washing in PBS, sections were incubated with a swine anti rabbit secondary antibody conjugated with TRITC. After washes, sections were covered with 50 % glycerol and stainings were visualised with a fluorescence microscope.

### Retinal morphometry

Quantitative retinal morphometry was carried out in retinal digest preparations as described previously [21]. In brief, eyes were fixed in 4 % formalin for 48 h, then retinas were dissected and incubated in aqua bidest for 30 min. Subsequently, the retinas were digested in 3 % trypsin dissolved in 0.2 M Tris-HCl (pH 7.4) for 3 h at 37°C. After washing by dropping water, the isolated retinal vasculatures were dried on objective slides and stained with PAS and hematoxylin. Quantitative analysis was achieved by counting endothelial cells and pericytes in a circular area of the middle 1/3 of the retina. Endothelial cells and pericytes were recognized by the shape of the nuclei, locations on capillaries and intensity of nuclear hematoxylin staining. Totally, 10 random areas were quantified using an imaging analyzing system (CUE-2, Olympus Opticals, Hamburg, Germany), and the numbers were normalized to retinal capillary area. Segments of acellular capillaries were quantified in 10 random areas by using an integration ocular, and the numbers of acellular capillary segments were normalized to mm^2^ retinal area. The analysis was performed in a masked fashion.

### Western Blot

Retinal proteins were extracted in a lysis buffer containing 125 mM NaCl, 10 mM EDTA, 25 mM Hepes, 10 mM Na3VO4, 0.5 % deoxycholic acid, 0.1 % SDS, 1% Triton-X-100 and cocktail of protein inhibitors. The lysate was homogenized by passing through 20-, 22-, 25- and 27-gauge needles at least 6 times. After centrifugation at 10000 rpm/min, the supernatant was collected and protein concentration was measured by Bradford assay. Protein extracts (10 microgram) were denatured by adding 5 % beta-mercaptoethanol and boiled for 5 min at 100°C. The proteins were separated by a 10 % SDS gel and transferred to PVDF membrane. Membranes were treated with 5 % non-fat milk for 1 h at room temperature. The primary rabbit anti GFAP (Santa Cruz Biotechnology) and the secondary swine anti rabbit IgG conjugated with horseradish peroxidase (DakoCytomation, Hamburg, Germany) were incubated over night and 1 h, respectively. For immunodetection, enhanced chemiluminescence kit (PerkinElmer LAS, USA) was used and the blots were exposed to Amersham Hyperfilms (Amersham, Buckinghamshire UK).

### RNA isolation and quantitative PCR

Retinal RNA was isolated from individual retinas homogenized in 1 ml Trizol reagent (Invitrogen, Karlsruhe, Germany) at 4°C according to the manufacturer's instructions. Then, RNA was reverse transcribed with QuantiTect Reverse Transcription kit (Qiagen GmbH, Germany) and subjected to Taqman analysis using the Taqman 2xPCR master Mix (Applied Biosystems, Weiterstadt, Germany). The expression of genes was analysed by the 2^−ΔΔCT^ method using beta-actin as housekeeping control. All primers and probes were purchased from Applied Biosystems (Weiterstadt, Germany). Beta-actin: Rn 00667869_m1: VEGF: Rn 00582935_m1; FGF2: Rn 00570809_m1; CNTF: Rn 00755092_m1; NGF: Rn 01833872_m1.

### Statistical Analysis

Data are presented as mean ± SD unless otherwise stated. Data were evaluated by analysis of variance (ANOVA) with Bonferroni posttests to show differences between the groups. A value of p<0.05 was considered as statistically significant.

## Results

### Neuronal changes occur during the first month in TGR rats

**Figure 1:**
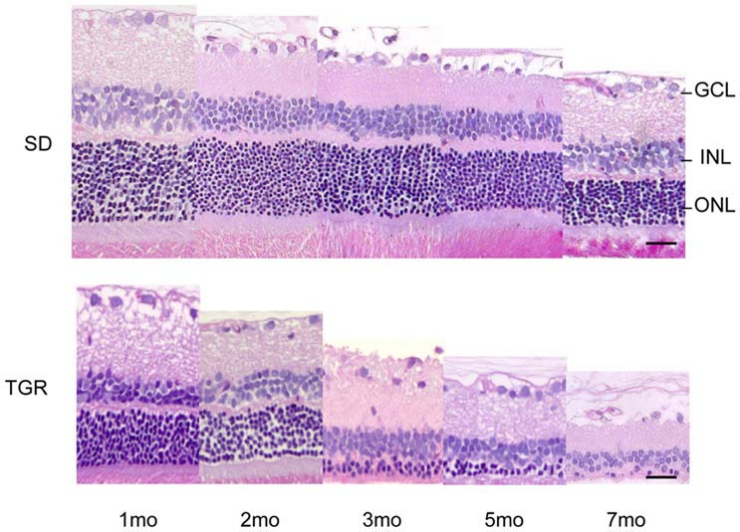
Illustration of histological assessment for neuronal changes in TGR rats from 1 to 7 months. Representative images showing progressive degenerating photoreceptor cells in the TGR rats compared to age-matched SD rats. Note that the SD rats have obvious photoreceptor cell loss during ageing. n = 3−8. Upper: SD rats; lower: TGR rats. GCL: ganglion cell layer; INL: inner nuclear layer; ONL: outer nuclear layer. Scales: 25 µm.

Truncated polycystin-2 is localized in the connecting part of the outer and inner segments of the photoreceptor cells. Therefore, we anticipated the primary damage in neuronal cells of the retina. Thus, we studied histological changes in the retina in a time course at 1, 2, 3, 5 and 7 months in paraffin sections. As illustrated in Fig. 1, retinal thickness was progressively reduced in TGR retinas compared to control SD retinas. Quantitative analysis summarized in Table 1 verified these observations. Thickness of the inner and outer nuclear layer and the whole retina in SD retinas from 1 to 7 months decreased significantly (p<0.05). Number of cells in the inner and outer nuclear layer reduced significantly (p<0.05), especially in 7-month SD retinas (p<0.001), demonstrating a spontaneous age-dependent change in SD retinas [22]. In TGR rats, the outer nuclear layer was significantly thinner than that in SD rats at the first month (p<0.05), and a constant significant decrease in thickness was found in the outer nuclear layer after second month compared to age-matched control SD rats. The ONL was reduced by 17% (p<0.05), 28% (p<0.05), 50% (p<0.001), 66% (p<0.05) and 68% (p<0.05) of age-matched SD rats at 1, 2, 3, 5 and 7 months of age, respectively. The whole retinas were significantly thinner in TGR retinas after 3 months of age. It was thinner by 15% (p>0.05), 20% (p>0.05), 33% (p<0.001), 31% (p<0.05) and 70% (p<0.05) of age-matched control SD rats at 1, 2, 3, 5 and 7 month, respectively. Furthermore, TGR retinas showed significantly less numbers of cells in the outer nuclear layer in the first month and a progressive decline with age compared to the control SD rats. Lost cells in the ONL reached 31% (p<0.001), 47% (p<0.001), 60% (p<0.001), 86% (p<0.01) and 97% (p<0.01) of age-matched control cells in the ONL at 1, 2, 3, 5 and 7 months of age, as related to each other. There was also loss of the inner nuclear cells in TGR retinas in the first month compared to SD retinas, but maximal loss of cells in the INL was only 24% of age-matched controls at the second month. No changes in thickness and cell numbers were observed in the ganglion cell layer. The neuronal changes occurred, therefore, predominantly in the outer nuclear layer. In TGR retinas at 7 months, although approximately 30 % of whole retinas were maintained, there were only few cells, probably 3 % of cells in the outer nuclear layer survived in comparison with the age-matched SD retinas. The outer nuclear layer had been reduced to loosely dotted single cells at 7 months. These data indicate that the incipient damage in TGR retinas is in the photoreceptor cells initiated during the first month of life and progresses over time.

**Table 1:** Thickness and cell number of TGR retinas.

	Age	GCL	INL	ONL	whole retina
Thickness	1mo SD	10.1±2.5	30.1±6.7	50.4±6.9	187.3±32.0
	2mo SD	9.7±3.3	23.1±5.7^#^	47.0±12.7	169.0±37.0^#^
	3mo SD	8.1±0.9	21.7±2.1^#^	40.3±5.1^#^	155.5±14.1^#^
	5mo SD	7.2±0.3	17.0±3.9^#^	33.6±5.7^##^	136.3±18.0^#^
	7mo SD	7.7±1.5	14.4±6.1^##^	27.8±9.5^##^	111.1±38.9^#^
	1mo TGR	9.9±0.9	20.5±2.6*	41.9±1.3*	158.4±8.7
	2mo TGR	7.4±1.0	21.3±1.7	34.0±5.0*	135.5±14.0
	3mo TGR	6.9±0.7	17.4±1.9	20.0 ±7.9^***^	104.0±17.1***
	5mo TGR	7.4±0.9	18.1±2.1	11.4±7.5*	93.6±18.8*
	7mo TGR	5.4±1.5	7.4±6.5	8.8±7.0*	32.9±29.3*
Cell number	1mo SD	2.9±1.0	34.7±4.8	139.9±13.5	
	2mo SD	2.3±0.4	28.8±3.7^#^	123.1±15.3^#^	
	3mo SD	2.2±0.3	27.9±2.9^#^	110.3±15.2^##^	
	5mo SD	2.0±0.7	22.8±3.1^##^	99.7±18.4^###^	
	7mo SD	1.3±0.1	16.7±1.0^###^	78.7±8.5^###^	
	1mo TGR	2.9±0.5	26.9±3.5*	96.3±11.3***	
	2mo TGR	2.2±0.4	21.9±3.2**	64.8±10.1***	
	3mo TGR	2.0±0.5	22.7±2.9**	43.4±17.7***	
	5mo TGR	1.7±0.4	20.7±0.8	13.5±6.0**	
	7mo TGR	1.6±0.3	18.1±5.5	2.3±2.1***	

The thickness and cell number of the TGR rats are mainly decreased in the outer nuclear layer. Note that there are only few cells in the outer nuclear layer at 7 months in the TGR rats. GCL: ganglion cell layer; INL: inner nuclear layer; ONL: outer nuclear layer. n = 3−8. #, ##, ### p<0.05, 0.01, 0.001, respectively, vs 1-month SD rats. *, **, *** p<0.05, 0.01, 0.001, respectively, vs age-matched SD rats.

### Retinal degeneration in TGR rats is the result of neuronal apoptosis

**Figure 2:**
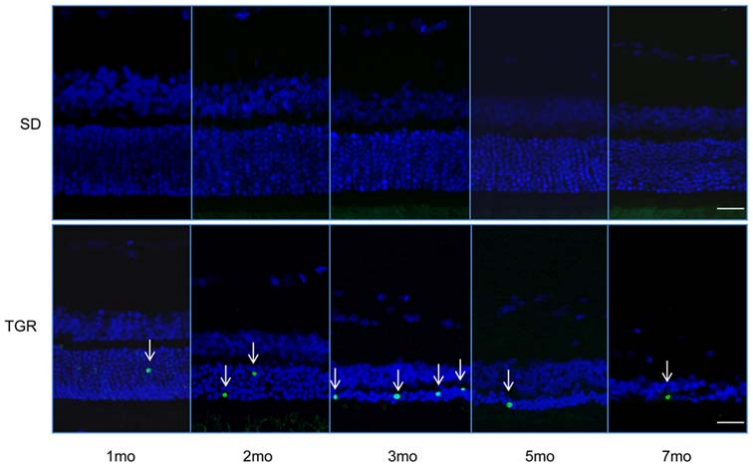
Apoptosis of photoreceptor cells in TGR rats from 1 to 7 months. Apoptosis occurs since the first month, peaks in the third month. Even in the 7-month retinas there are still apoptotic photoreceptor cells to be detected. No apoptotic cells are detectable in the SD rats. Upper panel: SD, lower panel: TGR. Arrows indicate apoptotic cells occurring in the photoreceptor cells in the outer nuclear layer. Scales: 25 µm.

To determine whether retinal degeneration is a result of neuronal apoptosis, TGR retinas at 1, 2, 3, 5 and 7 months were evaluated using terminal transferase dUTP nick-end labeling as described in materials and methods. As shown in Fig. 2, apoptotic cells were detected in the outer nuclear layer of TGR retinas, but no apoptotic cells were recognized in SD control retinas. The onset of apoptosis was observed during the first month of age and increased over time achieving a maximum at 3 months. There were a few apoptotic cells observed in the outer nuclear layer of the TGR retinas at 5 and 7 months of age. The localization and abundance of apoptotic cells in the TGR retinas from 1 to 7 months suggest that neuronal degeneration in TGR retinas is the result of increasing apoptosis in the photoreceptor cells where cilia protein is defective in TGR retinas.

### Functional defects of retinal neurons

**Figure 3:**
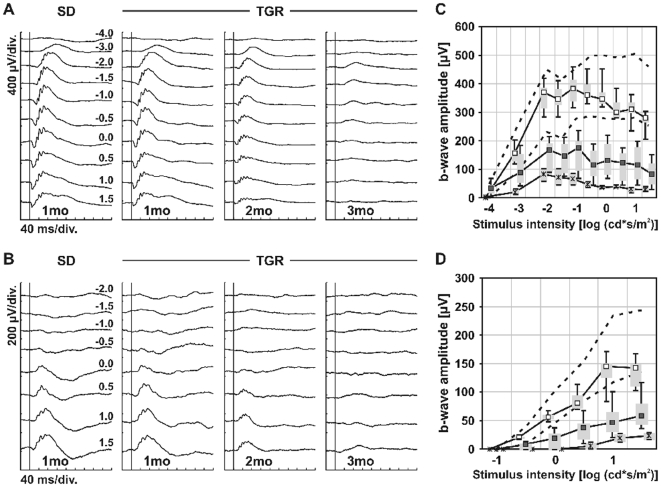
Retinal function of TGR rats. ERG age series in TGR rats. ERGs were recorded under (A) scotopic (dark-adapted) and (B) photopic (light-adapted) conditions from TGR rats 1, 2, and 3 months after birth. ERG responses from a 1-month-old SD rat are shown in the left column. (C) Scotopic and (D) photopic b-wave amplitudes from SD at 1 month and TGR at 1 (□), 2 (▪), 3 (*) months as a function of the logarithm of the flash intensity. Boxes indicate the 25% to 75% quantile range, whiskers indicate the 5% and 95% quantiles, and the asterisk indicates the median of the data. The 5% and 95% quantile of the 1-month SD data are shown with the black dotted lines.

To assess alterations in retinal neuronal function of TGR rats, flash ERGs were recorded from 1-, 2- and 3-month-old TGR and SD rats under scotopic and photopic conditions (Figure 3). Under both conditions, retinal functions were normal in TGR rats at the age of 1 month despite the incipience of apoptotic neural cell loss described above. However, 2-month-old TGR rats showed a considerable reduction in amplitudes of both scotopic and photopic ERG responses, indicating alterations of both rod and cone system. The retinal functions of TGR rats were further reduced at 3 months after birth, whereas ERGs in 3-month SD rats were unaltered.

### Vasoregression starts at the second month in TGR rats

**Figure 4:**
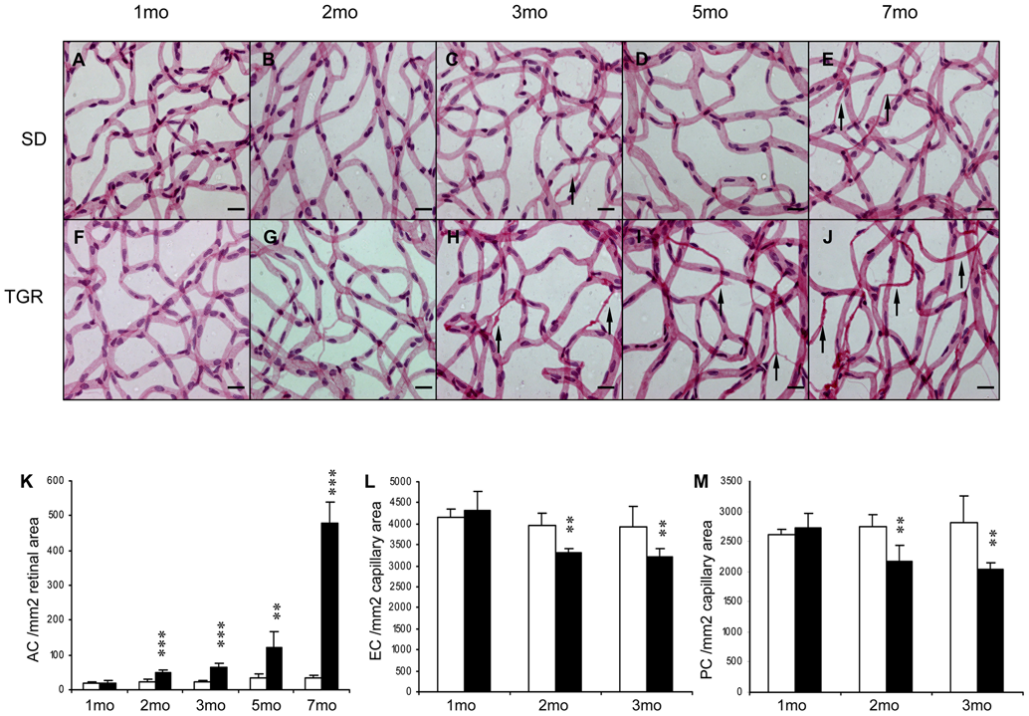
Vasoregression in TGR rats from 1 to 7 months. The TGR rats show a significant increase in the formation of acellular capillaries (arrows) and a decrease in the numbers of pericytes and endothelial cells from second month of life. A–E: SD rats, F–J: TGR rats. Quantitative analysis of formation of acellular segments (K), endothelial cell occupancy (L), and pericyte coverage (M). n = 4−7. *, **, *** p<0.05, 0.01, 0.001, respectively, vs age-matched SD rats. Scales: A–J: 20 µm.

To investigate the response of vasculature with regards to neuronal damages in TGR rats, we assessed the formation of acellular capillaries (ACs), the number of endothelial cells (ECs) and pericytes (PCs) in the retinal vasculature in retinal digest preparations. There was an exorbitant increase in the numbers of acellular capillaries in TGR retinas starting at two months of age. An increase in AC by 104% (p<0.001), 171% (p<0.001), 249% (p<0.01) and 1225% (p<0.001) in TGR rats compared to age-matched SD retinas was observed at 2, 3, 5 and 7 months, respectively (Fig. 4K). Notably, early vasoregression was mostly seen in midcapillary areas of the network suggesting that the initial degeneration starting in the deep capillary layer. Moreover, vascular abnormalities did not expand to arterioles and venules, and capillaries in proximity of larger vessels, i.e. those in the superficial layers, remained unaffected. Number of endothelial cells (Fig. 4L) and pericytes (Fig. 4M) were only quantifiable in TGR and SD retinas from 1 to 3 months because of the excessive formation of acellular capillaries resulted from markedly increased vasoregression after 5 months of age in TGR retinas. The number of endothelial cells and pericytes were comparable in 1-month TGR retinas and age-matched SD retinas (TGR: EC 4320±441/mm^2^ retinal capillaries, PC 2724±255/mm^2^ retinal capillaries; SD: EC 4164±197/mm^2^ retinal capillaries, PC 2619±90/mm^2^ retinal capillaries, p n.s.). Endothelial cells and pericytes were reduced significantly in TGR retinas at 2 (EC: −16%, p<0.01; PC: −21%, p<0.01) and 3 (EC: −19%, p<0.01; PC: −27%, p<0.01) months compared to age-matched SD retinas. Thus, vasoregression in TGR retinas ensues photoreceptor damage and death and neurodegeneration.

### Glia activation in TGR retinas

**Figure 5:**
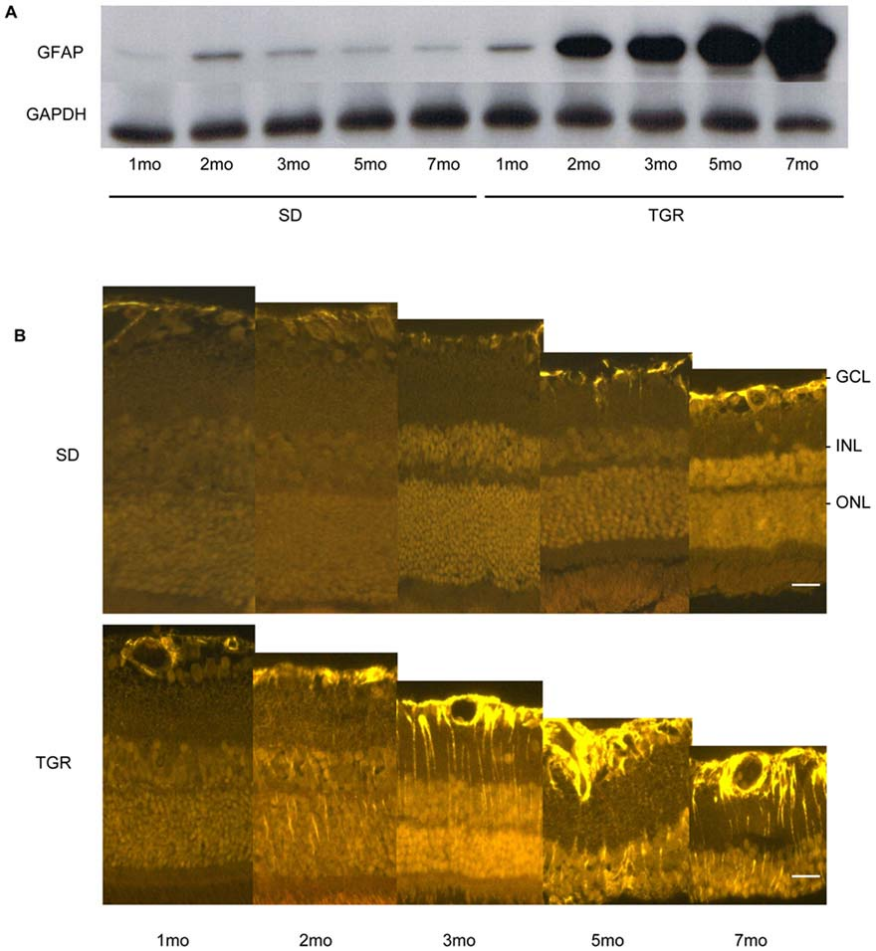
Glial activation in the TGR rats from 1 to 7 months. Representative demonstration of GFAP expression in Western blot (A) and immunofluorescence (B). In the TGR rats, GFAP is up regulated from the first month in astrocytes, and from the second month in Müller cells, while GFAP is weakly detectable in astrocytes in the SD rats. GCL: ganglion cell layer; INL: inner nuclear layer; ONL: outer nuclear layer. Scales: 25 µm.

Glial cell activation is common in response to retinal cell stress. To investigate glial activation due to neuronal damage in TGR retinas, we assessed the expression of glial fibrillary acidic protein (GFAP) in the retinas by western blot and immunofluorescence. As shown in Fig. 5, GFAP started to increase at one month of age in TGR retinas. Subsequently, the expression increased strongly over time. SD retinas showed generally low expression of GFAP in whole retinal lysate preparations at all time points observed. GFAP immunofluorescence staining of the TGR retinas displayed in astrocytes in the ganglion cell layer and in Muller cells throughout the entire retinas, while in control SD rats GFAP was weakly expressed in astrocytes in the ganglion cell layer.

### Induction of protective/survival factors in TGR retinas

**Figure 6:**
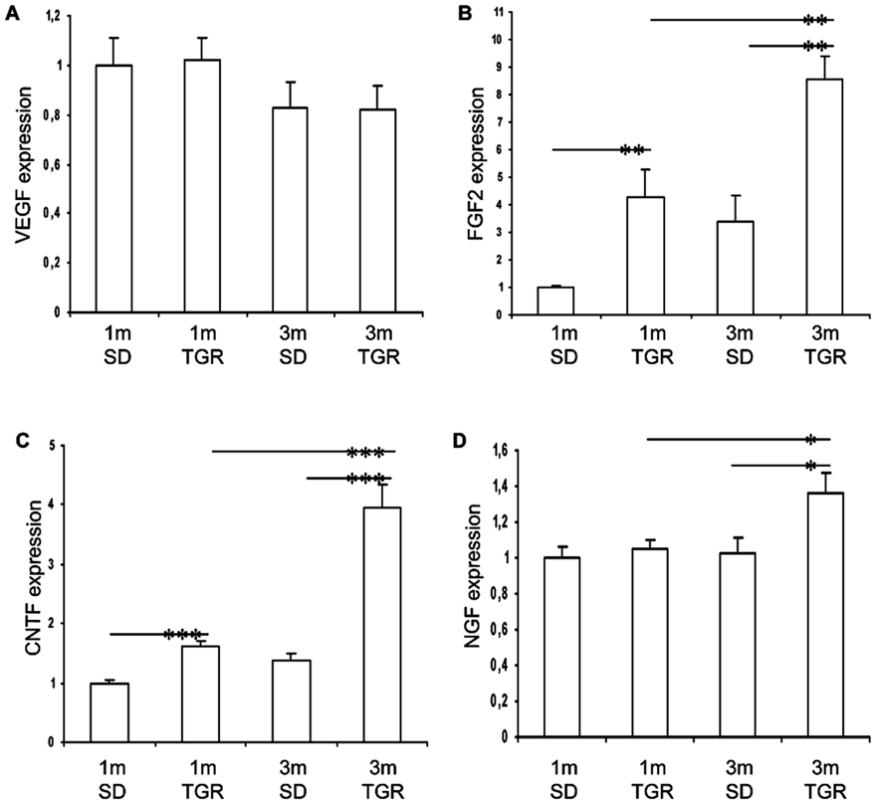
Induction of survival factors in TGR retinas during initiation of vasoregression. mRNA expression of VEGF (A), FGF2 (B), CNTF (C) and NGF (D) was evaluated by Taqman real time PCR. Expression levels in 1-month SD retinas are normalized to 1. Data are presented as mean ± SEM. n = 6. * p<0.05, ** p<0.01, *** p<0.001.

To investigate the implication of angioneurins in this model, we evaluated the expression of VEGF, FGF2, CNTF, and NGF during vasoregression. As vasoregression developed in the second month of life in TGR rats, to identify the genes involved in vasoregression, we compared 3-month old TGR and control rats with these groups at 1-month of age, i.e. at the onset of neurodamage. As shown in Fig. 6, VEGF was essentially unchanged during incipience of vasoregression (Fig. 6A). FGF2 and CNTF were markedly upregulated prior to vasoregression in 1-month old TGR retinas, suggesting that both factors were simultaneously stimulated by the ensuing neurodegeneration. In 3-month old TGR retinas, expression of both genes was further increased (Fig. 6B and C). In contrast, NGF was significantly induced at 3 months in TGR retinas compared to 3-month SD and 1-month old TGR retinas. These data demonstrate that VEGF is not involved in promotion of vessel survival in this setting, while FGF-2 and CNTF are responding early to the neuronal damage, whereas NGF, which is a strong vasculoprotective neurotrophin, responds only during the ensuing vasoregression process.

## Discussion

In this study, we identified a novel rat model with a defect in a cilia gene that mimics specific phenotypic changes which are likewise observed in diabetic retinopathy. In contrast to diabetic retinopathy, however, this model exhibits primary neuronal degeneration followed by vasoregression with a loss of capillary endothelial cells and pericytes during the second phase. Subsequent progressive impairment of retinal functions and concomitant glia activation and induction of neurotrophins in the retina of transgenic rat occur, which are at least in part similar to the pathologic evolution in diabetic retinopathy. While the expression of FGF2 and CNTF likely reflects a response to photoreceptor damage [23], NGF regulation may have a close link to retinal vasoregression.

An important finding in this study is the temporal attribution of neuronal damage with vasoregression. In TGR rats, photoreceptor cell death via apoptosis with a loss of cells in the inner nuclear layer develops during the first month prior to vasoregression which follows during the second month. Despite similarities of the neuronal changes in TGR rat with several animal models such as the rd/rd, rds mouse and Royal College of Surgeons (RCS) rat, and reported vascular alterations, specific differences exist regarding the onset and developmental dynamics of the photoreceptor death. For example, in the rd/rd mouse, rod cell death starts at the second week and is completed by the third week [24]. In this model, the vasculature starts to regress while still developing [14], [25]. Vascular changes are unknown in the rds mouse model with early onset at 2 weeks and slow progression of retinal degeneration up to 12 months. Moreover, late vascular changes due to the progressive loss of photoreceptors are reported from RCS rats [15], [26], but neither the temporal and spatial allocation nor an exact quantitation of vascular damage has been provided.

We observed both exponential vasoregression and pericyte loss in the TGR model. Vasoregression is also the hallmark of diabetic retinopathy in both, humans, and animal models [2]. Changes in pericyte coverage of microvessels, in endothelial cells and an increasing number of acellular capillaries of TGR retinas are qualitatively similar to those in experimental diabetic retinopathy [21]. Pericyte loss is an early and archetypical feature of diabetic retinopathy. In contrast to the diabetic model, in which pericyte loss appears to be causally linked to glucose-induced changes in angiopoietin-2 expression, the mechanism of pericyte loss in the TGR model remains yet to be determined. Neither glial activation which occurs prior to pericyte loss in the TGR model nor endothelial cell loss which starts in parallel with pericyte loss might be responsible, since key factors determining pericyte recruitment are not altered in TGR model. Angiopoietin-1 is produced by glial cells, and PDGF-B by endothelial cells. Neither of which are changed in the TGR model (unpublished data). Pericytes act as survival factors for established capillaries, and their loss may therefore be relevant in vasoregression [27], [28]. However, despite the similar degree of pericyte dropout in the TGR compared to the diabetic model, the degree of acellular capillaries differs substantially between the two models. Therefore, pericyte loss is unlikely to explain the enormous level of vasoregression in the TGR. The final cause for the exorbitant demise of vessels remains unclear, but it is obvious that the disturbed integrity of the neurovascular unit is responsible.

One factor relevant for proper retinal vessel function and neurovascular integrity, i.e. VEGF, does not respond to the neuronal damage in the TGR model. In contrast to other models of retinal degeneration such as the rho-/- mouse [29] in which VEGF is reduced, we did not find hypoxia in the TGR model, and consistent with the lack of hypoxia, VEGF was not regulated with progressive vascular regression. The absence of VEGF regulation is one of the discrepancies between the TGR and the diabetic retinopathy model in which VEGF is induced by hyperglycemia and the resultant increase in oxidative stress and advanced glycation endproducts formation. However, we also did not find a downregulation of VEGF as has been observed in some models of neurodegeneration, in which VEGF deficiency can impair neuronal survival.

Neurotrophic factors expressed in neuroglial cells of the retina, such FGF2, CNTF, and NGF, may play an important role in retinal neurodegeneration. NGF injection rescues photoreceptor degeneration in the RCS rat model of retinitis pigmentosa, involving secondary effects by other neurotrophins such as FGF2 and VEGF [30]. NGF also inhibits retinal degeneration in the C3H mouse [31]. In a hindlimb ischemia model, NGF induced angiogenesis, suggesting that NGF upregulation is protective against both, neurodegeneration and vascular regression [32]. Our previous data suggest that NGF treatment of diabetic rats prevents both, early neuroglial damage and the development of pericyte loss and vasoregression [33]. Our present data indicate that NGF is only upregulated after the onset of vasoregression, and after substantial neuronal cell loss has occurred.

In contrast, the two other neurotrophic factors studied, CNTF and FGF2, were upregulated prior to vasoregression and are found to be in close relationship with neurodegeneration. CNTF can delay photoreceptor degeneration in several models of genetic degeneration and ischemic injury. It is known that endogenous CNTF is upregulated in response to retinal injury, but the effect might be indirect (glia-mediated) rather than a direct effect, since the presence of CNTF-receptors on photoreceptors has not been unequivocally demonstrated. CNTF belongs to the CNTF/LIF group of cytokines. These have been extensively studied for their role in photoreceptor development. However, much less is known about the impact on vascular function. Recently, Kubota et al have demonstrated that LIF is involved in regulating microvessel density by regulating VEGF expression in mice [34]. LIF-/- mice had a denser capillary network with sustained tip cell activity, and despite resistance to hyperoxic vasoregression, they developed more neovascular tufts. These data suggest that there may be a link between the vasoregressive phenotype and increased expression of the CNTF/LIF family of cytokines in the retina of TGR. CNTF expression parallels that of FGF2 in our TGR model. FGF2 knockout mice develop photoreceptor degeneration suggesting that FGF2 plays an important role in photoreceptor development and survival. Multiple degenerative and injurious retina models yield upregulation of FGF2 suggesting the pleiotropic and essential role for FGF2 in survival of retinal cells. The lack of functional FGF2 could be a factor that causes the impaired protection of the diabetic retina from progressive vasoregression during the non-proliferative phase. Thus, in contrast to CNTF upregulation, FGF2 appears to be regulated as a response to injury type of tissue reaction in the TGR rat.

The Müller cell and astrocyte, which interconnect vessels and neurons through their end-foot processes, participate in neuroprotection and neuronal repair after injury [35]. Thus, increased GFAP expression as early as after 1 month suggests that glial activation occurs in response to neuronal degeneration in TGR rats. However, upregulation of GFAP is unspecific, as it occurs in various retinal injury models such as axotomy, retinal ischemia, retinal detachment and diabetic retinopathy. In contrast to the diabetic model, glial activation in the TGR model was not sufficient to induce VEGF, while bFGF upregulation is possibly the result of glial activation.

Mutations in cilia genes or defective cilia genes are associated with renal abnormalities, retinal degeneration, liver and respiratory diseases in patients [17], [18], [36]. In a previous study using the TGR rat, defective polycystin-2 gene was found in the region of connection cilia of outer and inner segments of rod and cone photoreceptors [16]. However, electron microscopy studies did not reveal any morphological cilia defects except photoreceptor degeneration in the TGR (unpublished data). The precise function of polycystin-2 in the retinal cilia remains unclear. Changes of polycystin-2 gene expression in the retinal cilia may lead to defective transport of functional proteins through this apparatus, although no change in the morphology of cilia in the TGR retina has been detected so far. The link between the genetic defect of polycystin-2 and the apoptosis of photoreceptors requires further investigations. Since the genetic makeup of the TGR rat has no human correlate, the model must not be considered as an animal model reflecting a specific human disease.

In summary, this study provides insight into the relationship between neurodegeneration, glial activation and vessel regression. Further evaluation of the molecular mechanisms involved in vasoregression in the TGR rat and comparative studies with models of vasoregression of diabetic origin supposedly yield novel targets for intervention of retinal vasoregression.
